# Chemical Profiling and Evaluation of Antioxidant and Anti-Microbial Properties of Selected Commercial Essential Oils: A Comparative Study

**DOI:** 10.3390/medicines4020036

**Published:** 2017-06-05

**Authors:** Ângelo Luís, Ana Paula Duarte, Luísa Pereira, Fernanda Domingues

**Affiliations:** 1 Centro de Investigação em Ciências da Saúde (CICS-UBI), Universidade da Beira Interior, Av. Infante D. Henrique, 6200-506 Covilhã, Portugal; afluis27@gmail.com (Â.L.); apcd@ubi.pt (A.P.D.); 2 Centro de Matemática e Aplicações (CMA-UBI), Universidade da Beira Interior, Rua Marquês d’Ávila e Bolama, 6201-001 Covilhã, Portugal; lpereira@ubi.pt

**Keywords:** essential oils, gas chromatography-mass spectrometry (GC-MS) analysis, antioxidant, anti-bacterial, anti-quorum sensing

## Abstract

**Background**: The last decades have seen an increased awareness by the scientific community of the extent of resistance to conventional antibiotics, particularly with respect to the emerging multidrug-resistant pathogenic microbes. Additionally, natural antioxidants have received significant attention among food professionals and consumers because of their assumed safety and potential therapeutic value. The aim of this work was to assess the antioxidant activities of eight selected commercial essential oils (EOs), together with the evaluation of their antibacterial and anti-quorum sensing properties. **Methods**: The chemical profiling of the EOs was performed using gas chromatography-mass spectrometry (GC-MS) analysis. The antioxidant properties of the EOs were evaluated using the 2,2-diphenyl-1-picrylhydrazyl (DPPH) free radical scavenging assay and by β-carotene bleaching test. Disc diffusion assays were employed to evaluate the anti-bacterial and anti-quorum sensing activities of the EOs. **Results**: It was observed that EOs from three *Eucalyptus* species are rich in eucalyptol. Generally, linalool is abundant in EOs from four *Lavandula* species. The oil of *Cymbopogon citratus* is the one with the best capacity to scavenge the DPPH free radicals and presented great antibacterial activity. **Conclusions**: The geographical origins of the plant species are determinant factors in the EO composition and in the corresponding biological activities.

## 1. Introduction

The last decades have seen an increased awareness by the scientific community of the extent of resistance to the conventional antibiotics, particularly with respect to the emerging of multidrug-resistant pathogenic microbes, as well as the appearance of unwanted side effects caused by inadequate and excessive use of the antibiotics. This resulted in a strong interest for the exploration of new sources of suitable anti-microbial drugs [1]. Plants have traditionally been the main origin of new compounds with potential anti-microbial properties that can be used by the pharmaceutical industry [1]. Additionally, natural antioxidants have gained significant attention among food professionals and consumers because of their assumed safety and potential therapeutic value [2].

Essential oils (EOs) present strong odor and flavor due to their complex chemical composition characterized by a mixture of volatile compounds that are biosynthesized in several plant parts (buds, flowers, leaves, stems, twigs, seeds, fruits, roots, wood, or bark) and stored in secretory cells, cavities, canals, epidermic cells or glandular trichomes [3]. EOs are valuable natural products used as feedstocks in perfumes, cosmetics, aromatherapy, phytotherapy, spices and nutrition [4]. Nowadays, approximately 3000 EOs are known, about 300 of which are commercially available [5]. The major constituents of EOs are aromatic and aliphatic compounds, most of them belonging to terpene/terpenoid and phenylpropanoid derivatives [5], which are characterized as low-molecular-weight aroma chemicals [5]. Generally, EOs are comprised of two or three major components in relatively high concentrations (20–95%) and other components present in trace levels [5].

*Eucalyptus* species (eucalypt) are well known medicinal plants because of their bioactive properties, which results in a great demand for their EOs that are routinely used not only by the pharmaceutical industry but also by the soap and cosmetic industries [6]. EOs of the *Lavandula* species (lavender) exhibit various biological activities, namely anti-microbial, antimutagenic, anti-inflammatory and analgesic properties [7]. *Rosmarinus officinalis* (rosemary) is the unique spice sold on the market as an antioxidant both in Europe and United States, commercialized in different types of formulations [8]. Berries of *Juniperus communis* (juniper) are commonly used in flavorings, perfumes and for aromatizing alcoholic beverages like gin [9]. In folk medicine, these berries are used because of their diuretic, antiseptic, stomachic, and carminative properties [9]. The EOs from needles, berries or wood of juniper have been subjected of several studies [9]. *Cymbopogon citratus* (lemongrass) is a prominent medicinal plant, that is frequently explored in traditional medicine for the treatment of various disorders [10]. Popularly, the aqueous infusion of this plant species is named “*abafado*” by Portuguese people, and was considered to have bioactive efficiency in the treatment of nervous and gastrointestinal ailments [10].

The aim of this work was to assess the antioxidant activities of eight commercial selected EOs, together with the evaluation of their antibacterial and anti-quorum sensing properties. The chemical profiling of the EOs was also performed using gas chromatography-mass spectrometry (GC-MS) analysis. The commercial selected EOs were obtained from eight plant species: *Eucalyptus citriodora*, *Eucalyptus smithii*, *Eucalyptus staigeriana*, *Lavandula angustifolia*, *Lavandula officinalis*, *Rosmarinus officinialis*, *Cymbopogon citratus* and *Juniperus communis*. It was also intended to compare the chemical composition and the bioactivities of the EOs of the same genus, and those from different geographic origins.

## 2. Materials and Methods 

### 2.1. Essential Oils

EOs were acquired commercially in a local pharmacy (Absolute Aromas, Covilhã, Portugal), except for *Lavandula officinalis* (Croatia) EO, which was bought in a local store in Dubrovnik (Dubrovnik Souvenirs, Croatia). All the oils were obtained by hydrodistillation from the fresh or dried aerial parts of the plant species. The EOs are produced and certified as biological products to be used in humans. Table 1 details the characteristic data of all the EOs considered in this study.

### 2.2. Gas Chromatography-Mass Spectrometry (GC-MS) Analysis

EOs were analyzed in an Agilent 7890A gas chromatograph coupled with an Agilent MS220 ion trap spectrometer (Agilent, Santa Clara, CA, USA). The identification of the compounds was performed using a time database (Gaithersburg, MD, USA) that was tested against the NIST12 mass database (Gaithersburg, MD, USA). An Agilent VF50 column (30 m length, 0.25 mm diameter, and 0.25 µm thickness) (Agilent, Santa Clara, CA, USA) was used. The assays were started at a temperature of 50 °C for 5 min; afterwards, the temperature was raised to 180 °C at a rate of 2 °C/min and this final temperature was maintained for 30 min. The temperature of the injection port and transfer line was set at 230 °C. The split injection mode (ratio 1:20) was adopted, with helium as the carrier gas at a constant flow rate of 1 mL/min. The mass spectrometer was operated in the mode of electron ionization with an electron energy value of 10 µA. The identification of the compounds was confirmed based on their retention indexes and their mass spectra which were compared to those obtained from available libraries. The analysis was repeated two times.

### 2.3. Antioxidant Activity Evaluation

#### 2.3.1. The 2,2-diphenyl-1-picrylhydrazyl (DPPH) Free Radical Scavenging Assay

The antioxidant activity of the EOs was evaluated by the 2,2-diphenyl-1-picrylhydrazyl (DPPH) free radical scavenging assay, previously implemented [11]. Briefly, 0.1 mL of several concentrations of the EOs (100, 75, 50, 25, 10 and 5% v/v) were mixed with 3.9 mL of three DPPH methanolic solutions with different concentrations: 0.2000, 0.1242 and 0.0800 mM, which were prepared by dissolving 39.4, 24.5 and 15.8 mg of DPPH (Sigma–Aldrich, St. Louis, MS, USA) in 500 mL of methanol (Fluka, Milwaukee, WI, USA), respectively. The negative control was composed by 0.1 mL of methanol and 3.9 mL of each DPPH solution. The reference compound used as positive control was gallic acid (Sigma–Aldrich, St. Louis, MS, USA). After the incubation period (90 min) at room temperature in the dark, the absorbance was measured at 517 nm using a spectrophotometer (Helios–Omega, Thermo Scientific, Waltham, MA, USA). The radical scavenging activity was calculated using the following equation:

(1) $$\%\;Inhibition=\;\frac{A_{control}-A_{sample}}{A_{control}}\times 100,$$

where *A_control_* was the absorbance of the control sample and *A_sample_* was the absorbance in the presence of the EOs. The IC_50_ (concentration providing 50% of inhibition), was determined using a calibration curve in the linear range of the graphic by plotting the EOs concentration versus the corresponding scavenging effect (% Inhibition). The antioxidant activity was expressed as the antioxidant activity index (AAI), calculated as follows:

(2) $$AAI=\frac{final\;concentration\;of\;DPPH\;in\;the\;control\;sample}{IC_{50}}.$$

Thus, the AAI was determined considering the mass of DPPH and the mass of the EO in the reaction, resulting in a constant for each EO, independent of the concentration of DPPH or EO used. The AAI allowed the following classification for the antioxidant activity of the EOs: poor (AAI ≤ 0.5), moderate (0.5 < AAI ≤ 1.0), strong (1.0 < AAI < 2.0), or very strong (AAI ≥ 2.0) [12]. Assays were carried out in triplicate and all DPPH solutions were prepared daily.

#### 2.3.2. β-Carotene Bleaching Test

The optimized protocol of the β-carotene bleaching test was also employed to evaluate the antioxidant properties of the EOs [12]. A volume of 500 μL of a β-carotene (Sigma-Aldrich, St. Louis, MS, USA) solution (20 mg/mL in chloroform), was added to 40 μL of linoleic acid (TCI Europe N.V., Zwijndrecht, Belgium), 400 μL of Tween 40 (Riedel-de Häen, Seelze, Germany) and 1 mL of chloroform (Scharlab, Barcelona, Spain). The chloroform was then evaporated under vacuum (45 °C), and 100 mL of oxygenated distilled water was added to the mixture to form an emulsion. Five milliliters of this emulsion was pipetted into test tubes containing 300 μL of the methanolic dilutions of the EOs (5, 3.75, 2.5, 1.25, 0.5 and 0.25% v/v). The negative control consisted of 5 mL of the emulsion and 300 μL of methanol, and the reference antioxidant butylated hydroxytoluene (BHT) (Fluka, Milwaukee, WI, USA) was used as positive control. Finally, the tubes were shaken and placed at 50 °C in a water bath for 1 h. The absorbances of the samples were measured at 470 nm, using a UV-Vis spectrophotometer (Helios–Omega, Thermo Scientific, Waltham, MA, USA), against a blank containing an emulsion without β-carotene. The measurements were carried out in triplicate at the initial time (*t* = 0 h) and at the final time of incubation (*t* = 1 h). The antioxidant activity of the EOs was determined as the percentage of inhibition of β-carotene oxidation (% Inhibition) by the following equation:

(3) $$\%\;Inhibition=\frac{A_{sample}^{t=1h}-A_{control}^{t=1h}}{A_{control}^{t=0h}-A_{control}^{t=1h}}\times 100,$$

where A^t=1h^ is the absorbance of the sample or control at the final time of incubation, and A^t=0h^ is the absorbance of the control at the initial time of incubation [12].

### 2.4. Determination of Antibacterial Activity

#### 2.4.1. Bacterial Strains and Growth Conditions 

Ten bacterial strains were used for the antibacterial studies: five Gram-positive strains (*Enterococcus faecalis* ATCC 29212, *Bacillus cereus* ATCC 11778, *Listeria monocytogenes* LMG 16779, *Staphylococcus aureus* ATCC 25923 and a clinical isolate of methicillin-resistant *S. aureus*, MRSA 10/08); and five Gram-negative strains (*Salmonella typhimurium* ATCC 13311, *Pseudomonas aeruginosa* ATCC 27853, *Escherichia coli* ATCC 25922, *Klebsiella pneumoniae* ATCC 13883 and *Acinetobacter baumannii* LMG 1025). The reference strains were acquired from either the American Type Culture Collection (ATCC, Manassas, VT, USA) or the BCCM/LMG Bacteria Collection (Belgian Co-Ordinated Collections of Micro-organisms, Gent, Belgium). Stock cultures of the bacterial strains were prepared and kept with 20% glycerol (Himedia, Mumbai, India) at −80°C. All the strains were sub-cultured in brain-heart infusion agar (BHI) (Liofilchem, Roseto degli Abruzzi, Italy) 24 h before the antibacterial assays. 

#### 2.4.2. Disc Diffusion Assay

The antibacterial activity of the EOs was evaluated by the disc diffusion assay, following the M2-A8 method as described by the Clinical Laboratory and Standards Institute (CLSI) for bacteria [13]. Inoculums were prepared by suspending bacteria in a sterile saline solution to a cell suspension of 0.5 McFarland (1 to 2 × 10^8^ colony-forming units/mL (CFU/mL)). Discs with a diameter of 6 mm were saturated with 10 µL of each EO. Then, the Müeller-Hinton agar (MHA) (Liofilchem, Roseto degli Abruzzi, Italy) plates were inoculated, allowed to dry, and the discs previously prepared were placed over the agar. The inoculated plates were incubated at 37 °C for 24 h. After the incubation, all the plates were visually checked for inhibition zones, the diameters being measured in millimeters. Each experiment was done independently three times [12].

### 2.5. Evaluation of Anti-Quorum Sensing Activity

The biomonitor strain *Chromobacterium violaceum* ATCC 12472 was used to assess the anti-quorum sensing activity of the EOs. The bacterial suspension of *C. violaceum* ATCC 12472 was obtained by overnight aerobic growth (30 °C, 250 rpm) in Luria-Bertani (LB) broth (Sigma–Aldrich, St. Louis, MO, USA).

#### Disc Diffusion Test

*C. violaceum* ATCC 12472 suspension was adjusted to an OD_620 nm_ of 1 and then seeded in LB agar (Pronadisa, Madrid, Spain) plates. Sterile discs (6 mm diameter) saturated with 10 µL of the EOs were placed over the plates and incubated (30 °C, 24 h). After the incubation period, the inhibition of the pigment production around the disc (a ring of colorless but viable cells) was measured. The lack of microbial growth indicates anti-microbial activity. Therefore, the bacterial growth inhibition was measured as diameter 1 (d_1_) in mm; while both bacterial growth and pigment inhibition were measured as total diameter 2 (d_2_) in mm. The quorum sensing inhibition (QSI), evaluated by the violacein pigment inhibition, was then determined by subtracting the diameter of bacterial growth inhibition (d_1_) from the total diameter (d_2_) (QSI = d_2_–d_1_) [14]. These experiments were performed in triplicate.

### 2.6. Statistical Analysis

Data were analyzed using the statistical program SPSS version 24 (IBM, Armonk, New York, NY, USA). The mean and standard deviation (SD) within triplicate assays were calculated for all cases. One-way analysis of variance (ANOVA) was undertaken to test for significant differences among means (*p* < 0.05 was considered significant).

## 3. Results

### 3.1. Chemical Profilling

The GC-MS analysis (Table 2, Table 3 and Table 4) resulted in the identification of more than 98% of the chemical volatile compounds present in the EOs. 

Table 2 lists the chemical composition of the EOs of *Eucalyptus* species. The major compound of *E. citriodora* oil is citronellal (78.15%). *E. smithii* oil is found to be rich in eucalyptol (81.71%), and *E. staigeriana* oil also presents eucalyptol (29.18%), in addition to bornyl formate (12.33%), and isobornyl formate (12.11%) as major constituents. It is known that in general EOs from the *Eucalyptus* species are rich in eucalyptol, as previously reported by our team [12] that characterized *E. globulus* and *E. radiata* EOs. Eucalyptol is a monoterpene that has been used for several purposes, including medicinal, flavor and fragrance [15]. This compound exhibits mosquito repellency, anti-tumor properties, and anti-inflammatory activity [15]. The oil of *E. citriodora*, also known as lemon-eucalypt, is mostly constituted by citronellal, a monoterpenoid, known to be the compound responsible for the intense lemon odor of this EO and also of EOs of *Citrus* species [16].

Table 3 indicates the results of GC-MS analysis for the *Lavandula* species EOs. The principal compound of the EO from *L. angustifolia* (Croatia) is linalool (66.83%), while for the same species grown in France, this compound only represents 37.31% of the chemical composition of the oil, being curcumin aldehyde (41.32%) the major component. Moreover, the oil of the same *Lavandula* species (*L. angustifolia*) grown at high altitude in France also presents a great amount of linalool (23.49%), but with linalyl formate (41.72%) being the most abundant compound. The main compounds of the other *Lavandula* species from Croatia (*L. officinalis*) are linalool (47.86%), linalyl formate (22.09%), and limonene (14.78%). Generally, linalool is abundant in EOs from *Lavandula* species. Linalool is a naturally-occurring terpene alcohol found in many plant species, presenting two enantiomers [17]. It has multiple commercial applications, the majority of which are linked to its pleasant scent (floral, with a touch of spiciness) [17]. The results now obtained, indicated that the major compound of the EOs obtained from the *Lavandula* species grown in Croatia is linalool, while in the EOs obtained from the French *Lavandula* species it is the second compound present; which suggest that the geographical origins of the plant species directly influences the chemical composition of the EOs.

Concerning the chemical composition of the EOs from *R. officinalis*, *C. citratus* and *J. communis*, the results of GC-MS analysis are presented in Table 4. *R. officinalis* major compounds are limonene (46.89%), and camphor (11.94%). High levels of limonene (59–88%) have been previously reported in individual oils from *Citrus* species [16]. The use of this terpene as a raw-material has been intensively investigated, and many new catalytic processes were reported affording valuable chemicals and polymers [18]. 

The main compounds of *C. citratus* EO are citronellyl formate (42.82%), and isobornbyl formate (33.52%).

The EO of *J. communis* is mostly consituted by *p*-mentha-1(7),8 diene (32.29%), and Δ-cadinene (11.28%). Cadinene is the chemical name given to various isomeric hydrocarbons that occur in a wide variety of plants known to produce EOs [9]. This name results from the wood of Cade juniper (*Juniperus oxycedrus* L.) that yields an EO from which cadinene isomers were isolated for the first time [19].

### 3.2. Antioxidant Properties

The antioxidant properties of the EOs, measured by the DPPH free radical scavenging assay and by the β-carotene bleaching test are summarized in Table 5.

The oil of *C. citratus* is the one with the best capacity to scavenge the DPPH free radicals, presenting an IC_50_ value of 0.03 ± 0.01 (v/v), that together with a value of AAI of 183.37 ± 22.00 allows to classify this oil as having a very strong antioxidant activity. It is important to emphasize that the parameters of the antioxidant activity of this oil, calculated by the DPPH method, allow the statistical grouping of the other oils in only one group, since the values obtained for the *C. citratus* oil are extremely different from the others.

*E. staigeriana* oil is the oil with the greatest antioxidant activity, measured by DPPH method, which is followed by the oils of *E. citriodora* and *E. smithii*. Regarding the *Lavandula* species, the EOs of *L. angustifolia* grown in France are the ones with very strong antioxidant activity, in contrary to what is verified with the EO of the same species grown in Croatia, presenting moderate antioxidant activity.

Observing the results of β-carotene bleaching test, an indirect measurement of the inhibition of the lipid peroxidation by the EOs, it was possible to verify that the *Juniper* species oil is the one with the lowest value of IC_50_ (1.48 ± 0.03 v/v), indicating that this oil presents the best capacity to inhibit the lipid peroxidation chain reactions.

### 3.3. Antibacterial and Anti-Quorum Sensing Properties

The diameters of the inhibition zones obtained for several Gram-positive and Gram-negative strains of bacteria tested in contact with the EOs are presented in Table 6.

Generally, the Gram-negative strains are more resistant to the effect of the EOs, presenting lower diameters of inhibition zones. It is described that Gram-negative bacteria should be less susceptible, which is associated with the characteristics of the outer-membrane of these microorganisms that endows the bacterial surface with strong hydrophilicity, acting as a robust permeability barrier [20].

The EO of *C. citratus* stands out from the other oils in this study, since it presents great antibacterial activity, as demonstrated by the high inhibition zone diameters, which was verified for most of the bacterial strains tested.

Among *Eucalyptus* species oils, those with best antibacterial properties are *E. citriodora* and *E. staigeriana* oils. 

Regarding the *Lavandula* species EOs, all of them presented significant antibacterial properties. In addition, no correlation was found between the antibacterial properties and the geographical origins of the EOs.

The resistance and pathogenesis of the bacteria could be linked to the mechanisms of cell–cell communication, like quorum sensing, which is initiated by the synthesis of small signaling molecules, than can be exchanged and perceived by the cells in the neighborhood [21]. This mechanisms allows Gram-positive and Gram-negative bacteria to regulate some physiological activities, such as virulence, competition amongst populations, conjugation, antibiotic production, motility, sporulation, and biofilm formation [21]. The signal molecules are involved in quorum sensing acyl homoserine lactone (AHL) [22]. A unique AHL detection system comprises the pigmented bacterium *C. violaceum*, which normally produces the purple compound violacein in response to the presence of N-hexanoyl homoserine lactone [22]. The inhibition of violacein production by *C. violaceum* is linked to the inhibition of the quorum sensing. In this sense, in the present work, the anti-quorum sensing activity of the EOs was also evaluated, using the biomonitor strain *C. violaceum*. The EO of *R. officinalis* presented the greatest capacity to inhibit the violacein production by *C. violaceum*, indicating its great anti-quorum sensing activity. Amongst *Eucalyptus* species EOs, *E. citriodora* is the only one with the capacity to inhibit the quorum sensing mechanisms in *C. violaceum*. With the exception of the *L. angustifolia* grown in Croatia, the EOs of *Lavandula* species were also able to block the quorum sensing process.

## 4. Discussion

Nowadays, EOs and some of their isolated compounds are used in various products such as cosmetics, household cleaning products, air fresheners, hygiene products, agriculture, or food, as well as in medicinal uses [5]. Therefore, the determination of the chemical composition of the EOs and the characterization of their biological activities are of major interest, both to academics and consumers. The present work aimed the assessment of the chemical profiling and the evaluation of the antioxidant and anti-microbial activities of selected EOs that are usually used due to their benefic properties to humans.

It was possible to observe that in general in the genus *Eucalyptus*, the major compound present in the EOs is eucalyptol, occurring in different concentrations. However, when looking at the antioxidant properties, it was noted that the oil with the greatest amount of this compound (*E. smithii* –81.71%), was the one with the weakest activity. These results indicate that the biological properties of the EOs are determined by the interaction of their components. This is, the compounds in the EOs may interact synergistically, conferring to the oils greater activities than those of the isolated compounds.

It was also noted that *Lavandula* species EOs are mainly constituted by linalool, but also that the geographical origins and the specific species influence the chemical composition of the EOs. The soil and climatic conditions available for the grown of the plant species are critical factors that influence the chemical composition of the EOs. In the present work, the EOs of *L. angustifolia* and *L. officinialis*, species of *Lavandula* were studied. Furthermore, two different countries of origin (Croatia and France) of those EOs were considered. The main compound of the EOs obtained from the *Lavandula* species grown in Croatia is linalool, and in the French *Lavandula* species it is the second compound, which indicates that the geographical origins of the plant species has a direct effect on the chemical composition of their EOs, having also repercussions on their bioactivities. The French *Lavandula* species EOs presented the best antioxidant activities when compared to those from Croatia. The EO with higher linalool content presents a lower antioxidant activity, in contrary to what is described for linalool and EOs with this compound, which are referred to as having relevant antioxidant activity [23,24]. This fact shows once again the importance of synergistic or antagonistic effects that might occur among the numerous components of the EOs. 

Concerning the anti-microbial properties of the EOs, the oil of *C. citratus* resulted in greatest inhibition of the growth of the bacterial strains under evaluation. It was also possible to note the presence of synergistic interactions among the compounds of the EOs that influenced the results obtained in the anti-bacterial test. 

Since the main challenge when using antibiotics is overcoming the appearance of resistance among the bacterial species, the inhibition of quorum sensing represents a highly attractive target for the development of novel therapeutics to prevent microbial infections [25]. The use of quorum sensing-based drugs to attenuate/combat bacterial pathogenicity rather than bacterial growth is particularly enticing with the emergence of increased antibiotic resistant bacteria [25]. Therefore, the inhibition of quorum sensing by the EOs was also evaluated, with *R. officinalis* oil having the most potential for use as a *quorum quenching* agent. 

An important issue to take into consideration when using EOs in humans, is that they cannot be toxic, for that reason, it is important to correlate the IC_50_ values obtained for the EOs under study with previous information about their potential toxic effects in humans. Llana-Ruiz-Cabello et al., 2015 [26] had reviewed the results obtained in vitro about the toxicological evaluation of several EOs and their main compounds [26]. In that work, the authors concluded that the most of the EOs in the bioactive concentrations, and also their main compounds, have been reported to be non-mutagenic and/or non-genotoxic to humans [26]. Moreover, it was stated that the main compounds of *R. officinalis* EO showed antimutagenic/antigenotoxic potential against several carcinogens [26]. Concerning linalool, a major compound found in several EOs, it is also commented that it exhibited antimutagenic potential [26]. Therefore, it can be inferred that in general and in the bioactive concentrations, EOs are safe to humans.

## 5. Conclusions

In conclusion, eucalyptol and linalool are the principal compounds of the EOs of *Eucalyptus* and *Lavandula* species, respectively. The geographical origins of the plant species are determinant factors in the EOs composition and in the corresponding biological activities.

The EO of *Cymbopogon citratus* presented remarkable antioxidant and antibacterial activities, which together with its pleasant odor, make this oil a promising natural agent to be used in both the pharmaceutical or food industry.

## Figures and Tables

**Table 1:** Origins data of essential oils (EOs) from aerial parts of different plant species.

Botanical Origins	Plant Family	Common Names of EOs	Geographical Origin
*Cymbopogon citratus* (DC) Stapf. ^1^	*Poaceae*	lemongrass	South Africa
*Eucalyptus citriodora* Hook. ^2^	*Myrtaceae*	lemon-eucalypt	China
*Eucalyptus smithii* R. T. Baker ^2^	*Myrtaceae*	eucalypt	Australia
*Eucalyptus staigeriana* F. Muell. ex Bailey ^2^	*Myrtaceae*	eucalypt	Australia
*Juniperus communis* L. ^1^	*Cupressaceae*	juniper berry	Bosnia
*Lavandula angustifolia* Mill. ^2^	*Lamiaceae*	lavender	Croatia
*Lavandula angustifolia* Mill. ^1^	*Lamiaceae*	lavender	France
*Lavandula angustifolia* Mill. ^1^	*Lamiaceae*	lavender	France (high altitude)
*Lavandula officinalis* Chaix ex Vill. ^3^	*Lamiaceae*	lavender	Croatia
*Rosmarinus officinalis* L. ^1^	*Lamiaceae*	rosemary	Tunisia

^1^ Supplied by Absolute Aromas; ^2^ Supplied by Base Formula; ^3^ Produced and bought in a local store in Dubrovnik (Dubrovnik Souvenirs).

**Table 2:** Chemical composition (% of compound) of the *Eucalyptus* species EOs.

*Eucalyptus citriodora*		*Eucalyptus smithii*		*Eucalyptus staigeriana*	
Compounds	Occurrence (%)	Compounds	Ocurrence (%)	Compounds	Occurrence (%)
Citronellal	78.15	Eucalyptol	81.71	Eucalyptol	29.18
Citronellol	5.55	α-Pinene	8.39	Bornyl formate	12.33
α-Caryophyllene	2.30	Limonene	3.28	Isobornyl formate	12.11
Eucalyptol	2.05	α-Terpineol	1.95	Terpinolene	6.73
β-Pinene	1.36	*o*-Cymene	1.12	β-Bourbonene	5.70
Citronellol acetate	1.33	γ-Terpinene	0.59	*Z*-Dimethoxycitral	5.68
α-Pinene	1.15	Terpinen-4-ol	0.49	α-Pinene	3.19
neo-Isopulegol	1.12	β-Pinene	0.42	*o*-Cymene	2.78
neoiso-Isopulegol	0.82	Myrcene	0.29	Terpinen-4-ol	2.47
α-Phellandrene	0.77	α-Phellandrene	0.28	Linalool	2.24
α-Cubebene	0.69	*trans*-Pinocarveol	0.18	β-Pinene	2.16
γ-Terpinene	0.62	Linalool	0.17	Neryl acetate	2.05
Longifolene	0.54	Δ-Terpineol	0.13	α-Terpineol	1.60
*o*-Cymene	0.51	Terpinolene	0.12	α-Phellandrene	1.47
Limonene	0.45	α-Caryophyllene	0.10	γ-Terpinene	1.38
Linalool	0.36	*p*-Cymen-8-ol	0.08	Nerol	1.12
Bergamal	0.20	Camphene	0.07	1-*p*-Menthene	1.01
*p*-Mentha-3,8-diene	0.20	*p*-Cymenene	0.05	*p*-Cymen-8-ol	0.81
Terpinolene	0.19	Citronellol acetate	0.05	Myrcene	0.78
α-Terpineol	0.18	α-Thujene	0.04	Citronellol acetate	0.76
Caryophyllene oxide	0.18	Aromadendrene	0.04	Carvone	0.74
β-Caryophyllene	0.17	Exo fenchol	0.04	Geranyl formate	0.59
Citronellic acid	0.13	Santolina triene	0.03	*p*-Mentha-1,5-dien-8-ol	0.41
Terpinen-4-ol	0.12	α-Gurjunene	0.03	*p*-Cymenene	0.36
Aromadendrene	0.09	α-Terpinene	0.03	*E*-β-Occimene	0.30
Myrcene	0.09	Geranyl acetate	0.03	α-Thujene	0.27
α-Thujene	0.07	Citronellal	0.03	α-Caryophyllene	0.22
Geranyl acetate	0.06	Fenchene	0.03	Camphene hydrate	0.22
Sabinene	0.06	*cis*-Linalool oxide	0.02	*Z*-β-Occimene	0.19
Nerol	0.06	Allo aromadendrene	0.02	α-Terpinene	0.15
Allo aromadendrene	0.06	*E*-β-Occimene	0.02	*p*-Mentha-3,8-diene	0.13
α-Terpinene	0.05	γ-Gurjunene	0.02	Spatulenol	0.11
*cis*-Rose oxide	0.05	β-Caryophyllene	0.02	α-Campholenal	0.09
Fenchene	0.02	Caryophyllene oxide	0.02	Globulol	0.09
Citronellyl formate	0.02	*trans*-Carveol	0.01	5-Hepten-2-one-6-methyl	0.07
*E*-β-Occimene	0.02	neo-Isopulegol	0.01	β-Caryophyllene	0.07
γ-Gurjunene	0.02	α-Campholenal	0.01	Sabinene	0.07
**TOTAL (37)**	**99.82**	Pinene oxide	0.01	Allo aromadendrene	0.06
		β-Gurjunene	0.01	Epiglobulol	0.06
		*cis*-Verbenol	0.01	2,6-Dimethyl-1,3,5,7-octatetraene *E,E*	0.06
		Verbenone	0.01	Aromadendrene	0.04
		Citronellic acid	0.01	Exo fenchol	0.04
		α-Terpinil acetate	0.01	α-Gurjunene	0.04
		**TOTAL (43)**	**99.98**	Camphene	0.03
				γ-Gurjunene	0.03
				*trans*-Carveol	0.02
				Germacrene *D*	0.01
				Fenchene	0.01
				*cis*-Rose oxide	0.01
				**TOTAL (49)**	**100.00**

**Table 3:** Chemical composition (% of compound) of the *Lavandula* species EOs.

*Lavandula angustifolia* (Croatia)		*Lavandula officinalis* (Croatia)		*Lavandula angustifolia* (France)		*Lavandula angustifolia* (High Altitude, France)	
Compounds	Occurrence (%)	Compounds	Occurrence (%)	Compounds	Occurrence (%)	Compounds	Occurrence (%)
Linalool	66.83	Linalool	47.86	Curcumin aldehyde	41.32	Linalyl formate	41.72
Camphor	8.92	Linalyl formate	22.09	Linalool	37.31	Linalool	23.49
Limonene	8.25	Limonene	14.78	α-Caryophyllene	4.53	α-Caryophyllene	7.05
Fenchyl acetate-exo	5.96	α-Terpineol	1.91	Borneol	2.14	*p*-Cymen-7-ol	5.04
Borneol	2.00	Terpinen-4-ol	1.37	*Z*-β-Farmesene	1.65	Terpinen-4-ol	4.02
Terpinen-4-ol	1.71	α-Caryophyllene	1.31	α-Terpineol	1.36	*Z*-β-Occimene	3.07
α-Terpineol	1.06	3-Octanone	1.13	Camphor	1.22	*E*-β-Occimene	2.85
α-Caryophyllene	0.71	Caryophyllene oxide	1.01	*Z*-β-Occimene	0.82	*Z*-β-Farmesene	2.13
β-Pinene	0.43	β-Pinene	0.86	3-Octanone	0.82	Borneol	1.07
*Z*-β-Occimene	0.37	*trans*-Linalool oxide (furanoid)	0.85	Caryophyllene oxide	0.81	Eucalyptol	0.97
α-Phellandrene	0.35	Eucalyptol	0.74	Linayl acetate	0.74	Caryophyllene oxide	0.78
α-Pinene	0.32	Borneol	0.67	*trans*-Linalool oxide (furanoid)	0.52	neo-Menyhol	0.77
*o*-Cymene	0.32	*cis*-Linalool oxide	0.61	Geranyl acetate	0.48	α-Curcumene	0.70
1-*p*-Menthene	0.30	α-Pinene	0.52	Eucalyptol	0.47	α-Terpineol	0.64
Linayl acetate	0.26	Camphor	0.45	Myrcene	0.44	epi-α-Cadinol	0.42
Isoborneol	0.24	epi-α-Bisabolol	0.39	*E*-β-Occimene	0.39	γ-Cadinene	0.39
*cis*-Linalool oxide	0.24	Hexyl-2-methyl butanoate	0.29	*cis*-Linalool oxide	0.36	Myrcene	0.38
Camphene	0.20	Geranyl acetate	0.27	Terpinen-4-ol	0.30	Camphor	0.35
*E*-β-Occimene	0.20	Sabinene	0.26	Camphene	0.30	Geranyl acetate	0.32
γ-Terpinene	0.12	*o*-Cymene	0.23	Neryl acetate	0.29	3-Octanone	0.29
3-Octanone	0.11	β-Caryophyllene	0.21	Germacrene *D*	0.27	*trans*-Linalool oxide (furanoid)	0.25
α-Terpinene	0.09	*Z*-β-Occimene	0.20	α-*trans*-Bergamotene	0.23	α-Terpinene	0.23
*Z*-β-Farmesene	0.09	Hexyl tiglate	0.18	β-Pinene	0.21	α-*trans*-Bergamotene	0.22
Sabinene	0.08	Crypton	0.17	3-Octanol	0.21	β-Caryophyllene	0.22
1-Octen-3-ol	0.07	Linayl acetate	0.16	Endo fenchol	0.20	α-Pinene	0.21
β-Caryophyllene	0.06	3-Octanol	0.16	Exo fenchol	0.20	*o*-Cymene	0.21
Geranyl acetate	0.05	α-*trans*-Bergamotene	0.14	Limonene	0.19	Isoledene	0.20
Germacrene *D*	0.05	α-Gurjunene	0.10	β-Caryophyllene	0.17	Limonene	0.19
Caryophyllene oxide	0.04	Curcumin aldehyde	0.09	2-Carene	0.17	Linayl acetate	0.17
Neryl acetate	0.04	*cis*-Linalyl oxide (pyranoid)	0.08	α-Pinene	0.15	Camphene	0.17
2-Carene	0.04	Camphene	0.08	epi-α-cadinol	0.15	1-Octen-3-ol	0.15
α-Thujene	0.04	β-Cubebene	0.07	γ-Cadinene	0.13	Endo fenchol	0.15
γ-Cadinene	0.04	*trans*-Pinocarveol	0.07	Δ-Cadinene	0.13	α-Thujene	0.11
Exo fenchol	0.03	Nerol	0.06	Hexanoic acid hexyl ester	0.13	β-Pinene	0.11
*trans*-2-Pinanol	0.03	γ-Cadinene	0.05	*o*-Cymene	0.11	2-Carene	0.10
*p*-Cymen-8-ol	0.03	α-Chamigrene	0.05	*cis*-Linalyl oxide (pyranoid)	0.08	β-Phellandrene	0.10
Geranyl propanoate	0.03	α-Thujene	0.04	Isobornyl acetate	0.07	Nerol	0.09
neo-Isopulegol	0.03	β-Sesquiphellandrene	0.04	α-Terpinil acetate	0.07	γ-Terpinene	0.09
epi-α-Bisabolol	0.03	Germacrene *D*	0.04	α-Gurjunene	0.07	Crypton	0.08
γ-Terpineol	0.02	β-Bisabolene	0.04	*p*-Cymene	0.06	Curcumin aldehyde	0.08
epi-α-Cadinol	0.02	α-*cis*-Bergamotene	0.04	*p*-Cymen-8-ol	0.06	*p*-Cymene	0.06
Camphene hydrate	0.02	epi-α-Cadinol	0.03	α-Famesene *E,E*	0.05	*p*-Cymen-8-ol	0.05
α-Gurjunene	0.02	*E*-β-Occimene	0.03	Neryl propianate	0.05	Sabinene	0.04
β-Cubebene	0.02	Isobornyl acetate	0.03	β-Bisabolene	0.05	Hexanoic acid hexyl ester	0.04
β-Sesquiphellandrene	0.01	β-Bourbonene	0.03	Geranyl propanoate	0.04	α-Phellandrene	0.03
α-Terpinil acetate	0.01	*p*-Cymen-8-ol	0.03	Terpinolene	0.04	Terpinolene	0.03
β-Bisabolene	0.01	2-Carene	0.03	β-Sesquiphellandrene	0.03	Carvacrol	0.03
α-Copaene	0.01	Spatulenol	0.02	β-Bourbonene	0.03	β-Bisabolene	0.03
Curcumin aldehyde	0.01	α-Curcumene	0.02	α-Campholenal	0.03	Δ-Cadinene	0.02
*p*-Cymene	0.01	Δ-Cadinene	0.02	neo-Isopulegol	0.02	Carvone	0.02
Δ-Cadinene	0.01	Neryl propianate	0.02	Sabinene	0.02	*cis*-Linalyl oxide (pyranoid)	0.02
Neryl propianate	0.01	Verbenone	0.02	α-Chamigrene	0.02	Epiglobulol	0.02
**TOTAL (52)**	**99.93**	*trans*-Carveol	0.01	α-Copaene	0.02	neo-Isopulegol	0.01
		*p*-Cymen-7-ol	0.01	α-Phellandrene	0.02	Verbenone	0.01
		α-Copaene	0.01	Pinene oxide	0.02	Geranyl formate	0.01
		Geranyl formate	0.01	α-Zingiberene	0.01	**TOTAL (55)**	**100.00**
		γ-Terpinene	0.01	γ-Terpinene	0.01		
		**TOTAL (57)**	**99.99**	α-Thujene	0.01		
				*cis*-Limonene oxide	0.01		
				*trans*-Pinocarveol	0.01		
				Verbenone	0.01		
				6-Camphenone	0.01		
				β-Gurjunene	0.01		
				Epiglobulol	0.01		
				p-Cymen-7-ol	0.01		
				**TOTAL (65)**	**99.87**		

**Table 4:** Chemical composition (% of compound) of the *R. officinalis*, *C. citratus* and *J. communis* EOs.

*Rosmarinus officinalis*		*Cymbopogon citratus*		*Juniperus communis*	
Compounds	Occurrence (%)	Compounds	Occurrence (%)	Compounds	Occurrence (%)
Limonene	46.89	Citronellyl formate	42.82	*p*-Mentha-1(7),8 diene	32.29
Camphor	11.94	Isobornyl formate	33.52	Δ-Cadinene	11.28
α-Pinene	10.65	Geraniol	3.37	*trans*-Muurola-4(14)1,5-diene	6.99
β-Pinene	5.19	Linalool	2.64	Limonene	6.19
Camphene	4.75	Geranyl acetate	1.79	α-Pinene	6.04
α-Caryophyllene	4.08	α-Caryophyllene	1.63	Δ-Sesquiphellandrene	5.70
Borneol	3.46	Caryophyllene oxide	1.49	*trans*-Cadina-1(6),4-diene	5.44
α-Terpineol	2.10	Terpinen-4-ol	1.36	α-*trans*-Bergamotene	3.79
*o*-Cymene	1.84	*m*-cymen-8-ol	0.94	Myrcene	3.02
Isobornyl acetate	1.37	α-Terpineol	0.78	Terpinolene	2.80
Myrcene	1.07	Camphene	0.75	Cubenol	2.04
Linalool	1.00	γ-Cadinene	0.74	Fenchene	1.44
Terpinen-4-ol	0.87	Eucalyptol	0.72	α-Chamigrene	1.42
γ-Terpinene	0.57	Camphene hydrate	0.63	α-Cadinol	1.38
β-Caryophyllene	0.51	γ-Terpineol	0.61	γ-Cadinene	1.22
α-Terpinene	0.36	Verbenone	0.59	Sabinene	1.05
Terpinolene	0.31	5-Hepten-2-one-6-methyl	0.54	α-Famesene *E,E*	0.84
Δ-Cadinene	0.29	Pinene oxide	0.49	α-Cubebene	0.59
2-Carene	0.28	4-Nonanone	0.41	Cubebol	0.52
α-Copaene	0.28	Limonene	0.37	β-Caryophyllene	0.44
α-Thujene	0.24	Myrcene	0.31	epi-Cubebol	0.43
γ-Muurolene	0.23	Δ-Cadinene	0.27	α-Caryophyllene	0.40
Caryophyllene oxide	0.21	Eugenol	0.23	Terpinen-4-ol	0.40
Tricyclene	0.18	β-Caryophyllene	0.23	α-Calacorene	0.33
γ-Cadinene	0.13	Camphor	0.22	β-Cubebene	0.32
Sabinene	0.11	Nerol	0.18	*p*-Mentha-2,4(8)-diene	0.31
α-Phellandrene	0.11	α-Pinene	0.13	*trans*-Cadina-1(2),4 diene	0.28
*trans*-Linalool oxide (furanoid)	0.09	Borneol	0.13	γ-Terpinene	0.24
α-Ylangene	0.08	*cis*-Rose oxide	0.11	α-Copaene	0.24
β-Bisabolene	0.08	α-Thujene	0.10	*p*-Cymen-8-ol	0.23
*trans*-Pinocarveol	0.07	α-*trans*-Bergamotene	0.10	*cis*-Muurola-4(15),5-diene	0.23
Exo fenchol	0.06	*Z*-β-Occimene	0.09	β-Pinene	0.20
α-Chamigrene	0.06	Citronellol acetate	0.08	Isobornyl acetate	0.16
1-Octen-3-ol	0.05	α-Ylangene	0.08	α-Cadinene	0.14
Aromadendrene	0.05	Geranyl formate	0.05	Santolina triene	0.14
α-Cubebene	0.04	*E*-β-Occimene	0.05	α-Terpinene	0.14
β-Gurjunene	0.04	2-Carene	0.04	α-Thujene	0.13
Camphene hydrate	0.04	Germacrene *D*	0.04	γ-Eudesmol	0.12
β-Sesquiphellandrene	0.03	α-Chamigrene	0.03	Butanoic acid hexyl ester	0.11
*p*-Cymen-8-ol	0.03	α-Copaene	0.03	Isoborneol	0.11
Germacrene *D*	0.03	α-Terpinil acetate	0.03	Linalyl formate	0.08
epi-Cubebol	0.03	*Z*-β-Farmesene	0.03	α-Terpineol	0.06
*E*-β-Occimene	0.03	*o*-Cymene	0.02	γ-Muurolene	0.05
1,3,6-heptatriene-2,5,6-trimetyl	0.02	Fenchyl acetate-exo	0.02	Linalool	0.05
α-Campholenal	0.02	*trans*-Linalool oxide (furanoid)	0.02	Caryophyllene oxide	0.04
*Z*-β-Farmesene	0.02	Terpinolene	0.01	Thymol methyl ether	0.04
Santolina triene	0.02	γ-Gurjunene	0.01	*o*-Cymene	0.03
β-Bourbonene	0.01	β-Pinene	0.01	*m*-Cymen-8-ol	0.02
α-Curcumene	0.01	Octanal	0.01	Dimethoxycitral-(*Z*)	0.02
γ-Gurjunene	0.01	γ-Terpinene	0.01	α-Terpinil acetate	0.02
3-Octanone	0.01	Sabinene	0.01	*p*-Mentha-1,5-dien-8-ol	0.02
α-*trans*-Bergamotene	0.01	**TOTAL (51)**	**98.88**	Tricyclene	0.02
iso-Isopulegol	0.01			2,6-Dimethyl-1,3,5,7-octatetraene *E,E*	0.01
**TOTAL (53)**	**99.98**			*Z*-β-Occimene	0.01
				Borneol	0.01
				**TOTAL (55)**	**99.62**

**Table 5:** Antioxidant properties of the EOs measured by two different methods.

Essential Oils/Reference Compounds	DPPH Free Radical Scavenging Assay			β-Carotene Bleaching Test
	IC_50_ ( v/v)	AAI	Strength of Antioxidant Activity	IC_50_ ( v/v)
*Eucalyptus citriodora*	0.51 ± 0.10 **a**	6.62 ± 1.04 **a**	Very strong	3.23 ± 0.05 **e**
*Eucalyptus smithii*	24.10 ± 0.05 **e**	0.19 ± 0.01 **a**	Poor	5.20 ± 0.10 **g**
*Eucalyptus staigeriana*	0.52 ± 0.11 **a**	10.45 ± 2.19 **a**	Very strong	1.94 ± 0.04 **b**
*Lavandula angustifolia* (Croatia)	3.39 ± 0.35 **b**	0.82 ± 0.09 **a**	Moderate	3.27 ± 0.04 **ef**
*Lavandula officinalis* (Croatia)	2.51 ± 0.32 **c**	1.65 ± 0.06 **a**	Strong	3.39 ± 0.09 **f**
*Lavandula angustifolia* (France)	1.70 ± 0.04 **d**	2.62 ± 0.10 **a**	Very strong	2.95 ± 0.03 **de**
*Lavandula angustifolia* (High altitude, France)	1.33 ± 0.07 **d**	4.04 ± 1.15 **a**	Very strong	2.79 ± 0.06 **d**
*Rosmarinus officinalis*	2.82 ± 0.02 **bc**	1.21 ± 0.36 **a**	Strong	3.20 ± 0.13 **e**
*Cymbopogon citratus*	0.03 ± 0.01 **a**	183.37 ± 22.00 **b**	Very strong	2.27 ± 0.04 **c**
*Juniperus communis*	1.18 ± 0.09 **d**	4.43 ± 1.10 **a**	Very strong	1.48 ± 0.03 **a**
Gallic acid	0.22 ± 0.01	22.77 ± 0.25	Very strong	-
BHT	-	-	-	3.58 ± 0.02

IC_50_—Concentration providing 50% of inhibition; AAI—Antioxidant activity index; BHT—Butylated hydroxytoluene; DPPH—2,2-diphenyl-1-picrylhydrazyl. Data expressed as means ± standard deviation (SD) of triplicate assays; Mean values in a column with different letters are significantly different (*p* < 0.05).

**Table 6:** Antibacterial and anti-quorum sensing properties evaluated as the diameter of inhibition zones (mm) of the EOs (10 µL/disc).

Bacterial Strains	Gram-Positive			Gram-Negative							QSI
Essential Oils	*Enterococcus faecalis* ATCC 29212	*Bacillus cereus* ATCC 11778	*Listeria monocytogenes* LMG 16779	*Staphylococcus aureus* ATCC 25923	MRSA 10/08	*Salmonella typhimurium* ATCC 13311	*Pseudomonas aeruginosa* ATCC 27853	*Escherichia coli* ATCC 25922	*Klebsiella pneumoniae* ATCC 13883	*Acinetobacter baumannii* LMG 1025	*Chromobacterium violaceum* ATCC 12472
*Eucalyptus citriodora*	12.70 ± 0.56 **ab**	40.30 ± 1.05 **c**	28.13 ± 3.44 **adef**	15.73 ± 1.57 **bc**	9.84 ± 1.01 **ab**	8.93 ± 0.40 **ab**	6.00 ± 0.00 **a**	12.72 ± 1.07 **abc**	12.59 ± 0.23 **ab**	12.20 ± 1.39 **a**	8.36 ± 0.93 **c**
*Eucalyptus smithii*	7.19 ± 0.62 **a**	9.78 ± 0.16 **a**	13.54 ± 1.12 **ab**	6.00 ± 0.00 **a**	6.65 ± 0.18 **a**	8.96 ± 0.74 **ab**	6.00 ± 0.00 **a**	11.57 ± 1.11 **ab**	9.12 ± 0.62 **a**	9.08 ± 0.76 **a**	0.00 ± 0.00 **a**
*Eucalyptus staigeriana*	23.08 ± 1.37 **d**	55.82 ± 1.68 **d**	33.67 ± 3.37 **f**	16.06 ± 1.97 **bc**	11.09 ± 1.24 **bc**	12.62 ± 0.53 **b**	7.68 ± 0.06 **b**	17.12 ± 0.52 **bc**	18.19 ± 1.82 **c**	20.26 ± 0.88 **b**	0.00 ± 0.00 **a**
*Lavandula angustifolia* (Croatia)	14.55 ± 0.69 **bc**	30.42 ± 2.67 **bc**	18.26 ± 2.38 **abc**	11.82 ± 1.38 **ab**	15.03 ± 1.13 **cd**	11.37 ± 1.00 **ab**	7.31 ± 0.37 **ab**	17.98 ± 1.58 **c**	16.67 ± 1.03 **bc**	19.30 ± 1.03 **b**	0.00 ± 0.00 **a**
*Lavandula officinalis* (Croatia)	20.29 ± 1.88 **cd**	17.73 ± 1.70 **a**	24.19 ± 3.15 **cde**	15.20 ± 1.28 **bc**	14.87 ± 1.03 **cd**	12.52 ± 0.86 **b**	7.40 ± 0.83 **ab**	17.78 ± 1.94 **c**	16.94 ± 0.43 **bc**	18.38 ± 0.66 **b**	4.84 ± 0.08 **b**
*Lavandula angustifolia* (France)	16.39 ± 2.10 **bc**	20.51 ± 2.79 **ab**	21.31 ± 1.68 **bcde**	22.43 ± 3.69 **c**	19.62 ± 0.13 **e**	10.58 ± 0.81 **ab**	7.38 ± 0.48 **ab**	13.33 ± 1.68 **abc**	15.40 ± 1.00 **bc**	41.64 ± 3.08 **d**	8.60 ± 0.56 **c**
*Lavandula angustifolia* (High altitude, France)	14.52 ± 1.47 **bc**	22.34 ± 3.95 **ab**	25.18 ± 1.73 **cdef**	18.61 ± 1.65 **bc**	16.05 ± 1.65 **de**	9.29 ± 0.83 **ab**	6.00 ± 0.00 **a**	10.60 ± 0.13 **a**	12.08 ± 0.53 **ab**	30.05 ± 1.55 **c**	5.01 ± 0.43 **b**
*Rosmarinus officinalis*	7.99 ± 0.74 **a**	10.75 ± 2.25 **a**	11.63 ± 1.62 **a**	20.10 ± 1.08 **c**	17.27 ± 1.88 **de**	9.87 ± 0.69 **ab**	7.27 ± 0.69 **ab**	11.54 ± 0.33 **ab**	13.58 ± 1.65 **abc**	33.84 ± 1.93 **c**	8.69 ± 0.81 **c**
*Cymbopogon citratus*	30.09 ± 1.20 **e**	75.60 ± 7.02 **e**	85.00 ± 0.00 **g**	45.90 ± 0.16 **d**	50.88 ± 0.50 **f**	12.32 ± 1.25 **b**	8.14 ± 0.09 **b**	17.44 ± 0.40 **bc**	17.20 ± 1.23 **bc**	45.51 ± 0.49 **d**	0.00 ± 0.00 **a**
*Juniperus communis*	11.07 ± 0.25 **ab**	16.41 ± 3.16 **a**	18.59 ± 1.35 **abc**	15.60 ± 2.39 **bc**	12.78 ± 0.57 **bc**	7.97 ± 0.81 **a**	7.12 ± 0.27 **ab**	8.92 ± 1.14 **a**	8.52 ± 0.40 **a**	12.40 ± 0.14 **a**	0.00 ± 0.00 **a**

QSI—quorum sensing inhibition; MRSA—methicillin-resistant *Staphylococcus aureus*; ATCC—American Type Culture Collection; LMG—Laboratory for Microbiology of the Faculty of Sciences of the Ghent University; Data expressed as means ± SD of triplicate assays; Mean values in a column with different letters are significantly different (*p* < 0.05).
